# The impact of valvular heart disease in patients with chronic coronary syndrome

**DOI:** 10.3389/fcvm.2023.1211322

**Published:** 2023-07-21

**Authors:** Mitchel A. Molenaar, Berto J. Bouma, Casper F. Coerkamp, Jelle P. Man, Ivana Išgum, Niels J. Verouden, Jasper L. Selder, Steven A. J. Chamuleau, Mark J. Schuuring

**Affiliations:** ^1^ Department of Cardiology, Amsterdam UMC Location Vrije Universiteit Amsterdam, Amsterdam, Netherlands; ^2^ Department of Cardiology, Amsterdam UMC Location University of Amsterdam, Amsterdam, Netherlands; ^3^ Amsterdam Cardiovascular Sciences, Amsterdam UMC, Amsterdam, Netherlands; ^4^ Department of Biomedical Engineering and Physics, Amsterdam UMC Location University of Amsterdam, Amsterdam, Netherlands; ^5^ Department of Radiology and Nuclear Medicine, Amsterdam UMC Location University of Amsterdam, Amsterdam, Netherlands; ^6^ Department of Cardiology, University Medical Center Utrecht, Utrecht, Netherlands

**Keywords:** coronary artery disease, valvular heart disease, prognosis, risk, mortality

## Abstract

**Background:**

The European Society of Cardiology 2019 Guidelines on chronic coronary syndrome (CCS) recommend echocardiographic measurement of the left ventricular function for risk stratification in all patients with CCS. Whereas CCS and valvular heart disease (VHD) share common pathophysiological pathways and risk factors, data on the impact of VHD in CCS patients are scarce.

**Methods:**

Clinical data including treatment and mortality of patients diagnosed with CCS who underwent comprehensive transthoracic echocardiography (TTE) in two tertiary centers were collected. The outcome was all-cause mortality. Data were analyzed with Kaplan-Meier curves and Cox proportional hazard analysis adjusting for significant covariables and time-dependent treatment.

**Results:**

Between 2014 and 2021 a total of 1,984 patients with CCS (59% men) with a median age of 65 years (interquartile range [IQR] 57–73) underwent comprehensive TTE. Severe VHD was present in 44 patients and moderate VHD in 325 patients. A total of 654 patients (33%) were treated with revascularization, 39 patients (2%) received valve repair or replacement and 299 patients (15%) died during the median follow-up time of 3.5 years (IQR 1.7–5.6). Moderate or severe VHD (hazard ratio = 1.33; 95% CI 1.02–1.72) was significantly associated with mortality risk, independent of LV function and other covariables, as compared to no/mild VHD.

**Conclusions:**

VHD has a significant impact on mortality in patients with CCS additional to LV dysfunction, which emphasizes the need for a comprehensive echocardiographic assessment in these patients.

## 1. Introduction

Chronic coronary syndrome (CCS) is characterized by stable atherosclerotic coronary plaques that build up over time. CCS affects more than 34 million adult Europeans and has a high mortality rate, despite advanced medical care and revascularization (1, 2).

The European Society of Cardiology (ESC) 2019 Guidelines on CCS recommend transthoracic echocardiographic (TTE) measurement of left ventricular (LV) function for risk stratification in all patients with CCS, as it is considered to be one of the strongest determinants of mortality (3–5). This is mainly based on the Coronary Artery Surgery Study (CASS) registry (5). This CASS registry was performed in the 1970s, a long time before introduction of modern therapies and revascularization techniques.

It is unclear whether other echocardiographic findings provide additional information about the prognosis of patients with CCS. In particular data regarding the impact of valvular heart disease (VHD) on the mortality of patients with CCS are scarce. It is estimated that VHD affects more than 18 million Europeans (6, 7) and accounts for 10 to 20% of all cardiac surgery procedures (8, 9). As both VHD and CCS share common pathophysiological pathways and risk factors, a better understanding of the impact of VHD on mortality in patients with CCS is needed. Therefore, we performed a study in CCS patients and investigated the impact of VHD on mortality.

## 2. Methods

### 2.1. Design and patient population

In this retrospective cohort study, patients diagnosed by the treating physician with CCS between 2014 and 2021 were consecutively selected from electronic health records of the Amsterdam University Medical Center (two tertiary centers), the Netherlands. CCS patients (≥18 years) who underwent comprehensive transthoracic echocardiography (TTE) one year before or after the outpatient visit were eligible for inclusion. For patients with multiple studies, the TTE closest to the outpatient visit date was selected. This retrospective cohort study was approved by the local institutional review board, who waived the need for written consent.

### 2.2. Data collection and echocardiographic measurements

Baseline, treatment and mortality in follow-up data of patients with CCS were collected from pseudonymized electronic health records and stored in a registry. Two-dimensional TTE with Doppler tissue imaging (Vivid 9, GE Vingmed Ultrasound AS, Horten, Norway; Philips Epiq, Philips Affiniti and Philips IE33, Philips Medical Systems, Best, The Netherlands) was performed and assessed by clinical technicians or cardiology residents according to recommendations of the European Association of Cardiovascular Imaging (10), ESC guidelines (11, 12) and standard operating procedure (13). TTE images were digitized and analyzed using vendor-specific software (GE EchoPAC, GE Vingmed Ultrasound AS, Horten, Norway; Xcelera, Philips Medical Systems, Best, The Netherlands; TomTec 2D Cardiac Performance Analysis, Munich, Germany).

The initial assessment of the valves was performed qualitatively by a clinical technician or cardiology resident. Semi-quantitative and quantitative measurements of stenosis or regurgitation were obtained if indicated, especially if clinical decisions were based on these findings (11, 12). The results were documented in a TTE report (10), which were overseen by dedicated imaging cardiologists who made corrections if needed to maintain accuracy and completeness.

The Simpson's method of disks was used to estimate the LV volume at both end-diastolic and end-systolic phase from apical four- and two-chamber views. This method involved tracing the endocardial border on 2D echocardiographic images of the left ventricle during these phases, dividing the tracing into multiple disks (slices), and summing their volumes to obtain the total LV volume. The LV ejection fraction (LVEF) was calculated by subtracting the LV volume at end-systole from the LV volume at end-diastole, and dividing it by the LV volume at end-diastole. LV dysfunction was defined as mild to severe abnormal LV function (LVEF <51% for male and <53% for female). Moderately and severely abnormal LV function were defined as a LVEF of 30%–41% and <30%, respectively (14).

For the purpose of this study, the LV function and severity of aortic stenosis (AS), aortic regurgitation (AR), mitral stenosis (MS), mitral regurgitation (MR), tricuspid regurgitation (TR), pulmonary stenosis (PS), and pulmonary regurgitation (PR) were stored in the registry. Patients were excluded if TTE image acquisition was of poor quality or incomplete due to missing assessment of LV function or VHD (10). Multivalvular disease was defined as regurgitation and/or stenosis in two or more heart valves. Patients were categorized based on the most severe valve condition among the valves, which means that patients with both moderate and severe valvular lesions were classified as having severe VHD. Impaired renal function was defined as an estimated glomerular filtration rate (eGFR) < 60 ml/min/1.73 m^2^. eGFR was calculated with the Chronic Kidney Disease Epidemiology Collaboration (CKD-EPI) creatinine equation (15). Obesity was defined as a body mass index of >30 kg/ m^2^.

### 2.3. Outcome

The clinical endpoint was all-cause mortality. The end of follow-up was defined as the last recorded contact with the tertiary center or the date of mortality.

### 2.4. Statistical analysis

Results were expressed as mean values with standard deviation (SD) for normally distributed data, and median with interquartile range (IQR) for not normally distributed data. Nominal or ordinal data were expressed with numbers and percentages. The Shapiro-Wilk test was used to test for normality. The one-way ANOVA or Kruskal-Wallis test was performed for between group comparisons of continuous data, as appropriate. A Pearson's Chi-Square test was performed for categorical variables.

Missing values were imputed by multiple imputation by chained equation (MICE) with a linear regression model, which was iteratively performed for 10 iterations. The degree of multicollinearity between variables was assessed with the variance inflation factor (VIF) (16). Variables with a VIF greater than 10 were either dichotomized, centered by subtracting the mean value, or omitted from multivariable analysis to account for their collinearity with other variables. Variables with a p-value of *p* ≤ 0.05 on univariable mortality analysis and time-dependent variables coronary revascularization and valve repair or replacement were entered into Cox proportional hazards (PH) models with backward selection procedure (*p* ≤ 0.05) based on the Akaike information criterion. Cox PH analyses were conducted to examine the association between predictor variables and mortality. Predictor variables with a prevalence of at least 5% among the patients were included in multivariable analysis. To evaluate the assumption of PH, Kaplan Meier curves were inspected and Schoenfeld residuals were calculated (17). Analysis was performed for the predictor variables VHD, the number of valves affected and the specific subtypes of VHD (AS, AR, MS, MR, TR, PS and PR).

Statistical analyses were performed in Python V3.8 and RStudio V.2022.07.0 (RStudio Team, Boston, MA) using R-version 4.1.3. (R Core Team, Vienna, Austria). *P *< 0.05 was considered statistically significant.

## 3. Results

### 3.1. Study population

A total of 2,845 patients with CCS were screened. Among them, 861 patients were not eligible for inclusion, resulting in a study population of 1,984 patients, as shown in Supplementary Figure S1. The proportion of missing data was 3%, as shown in Supplementary Table S1. The median age of the study population was 65 years (IQR 57–73), 59% were men and 26% had a history of myocardial infarction (Supplementary Table S2). The majority, 54%, had a history of hypertension and 59% of the patients presented with chest pain at the outpatient visit. A minority of the patients presented with dyspnea (31%). The most common reported secondary prevention therapies were antiplatelet therapy (61%), statins (60%) and beta-blockers (52%). A total of 505 patients (25%) had LV dysfunction, which was severe in 39 patients (2%), as shown in Supplementary Table S3.

### 3.2. Concomitant valvular heart disease

No/mild, moderate and severe VHD were present in 1,615 (82%), 325 (16%) and 44 (2%) patients, respectively (Supplementary Table S3). MR was most the common VHD (moderate MR: 176 patients, severe MR: 3 patients) in patients with CCS, followed by TR (moderate TR: 128 patients, severe TR: 16 patients), and AS (moderate AS: 64 patients, severe AS: 24 patients). Multivalvular disease was present in 84 patients (4%). Compared to patients within the group with no/mild VHD, patients with moderate or severe VHD were significantly older and had more often hypertension, atrial fibrillation or flutter, and chronic obstructive pulmonary disease (COPD), used more often cardiovascular medication (anticoagulants, renin-angiotensin system inhibitors, beta-blockers, and diuretics), had a lower eGFR, and more often LV dysfunction (Supplementary Tables S2, S3). Patients with moderate or severe VHD had more often surgical aortic valve replacement (SAVR) in medical history. Patients with no/mild VHD were more often a current or former smoker and had more often a family history of coronary artery disease. Chest pain was more often reported in patients with no/mild VHD, while dyspnea was more frequently reported in patients with moderate or severe VHD.

### 3.3. Follow-up

The median follow-up time of patients was 3.5 years (IQR 1.7–5.6). During the follow-up period, a total of 654 patients (33%) received revascularization by coronary artery bypass grafting (CABG) and/or percutaneous coronary intervention (PCI). Follow-up data are shown in Table 1. Patients with severe VHD received revascularization by CABG (11%) more often compared to patients with moderate (3%) or no/mild VHD (3%) during follow-up. Valve repair or replacement was performed in 39 patients (2%) during follow-up. Transcatheter aortic valve replacement (TAVR) was most often performed (26 patients) followed by SAVR (10 patients). One patient with mild VHD and 14 patients with moderate VHD received valve repair or replacement during follow-up due to multivalvular disease, increase in severity, or combined with CABG.

**Table 1:** Follow-up of study population.

	All patients (*n* = 1,984)	No/mild VHD (*n* = 1,615)	Moderate VHD (*n* = 325)	Severe VHD (*n* = 44)	*p*-value
Revascularization^a^, *n* (%)	654 (33.0)	517 (32.0)	120 (36.9)	17 (38.6)	0.165
CABG, *n* (%)	56 (2.8)	41 (2.5)	10 (3.1)	5 (11.4)	0.002
PCI, *n* (%)	622 (31.4)	492 (30.5)	116 (35.7)	14 (31.8)	0.179
Isolated or concomitant valve repair or replacement^b^, *n* (%)	39 (2.0)	1 (0.1)	14 (4.3)	24 (54.5)	<0.001
SAVR, *n* (%)	10 (0.5)	0 (0.0)	4 (1.2)	6 (13.6)	<0.001
TAVR, *n* (%)	26 (1.3)	1 (0.1)	8 (2.5)	17 (38.6)	<0.001
SMVR, *n* (%)	1 (0.1)	0 (0.0)	0 (0.0)	1 (2.3)	<0.001
TEER, *n* (%)	3 (0.2)	0 (0.0)	2 (0.6)	1 (2.3)	<0.001
STVR, *n* (%)	1 (0.1)	0 (0.0)	0 (0.0)	1 (2.3)	<0.001
SPVR, *n* (%)	0 (0.0)	0 (0.0)	0 (0.0)	0 (0.0)	NA
Outcome, *n* (%)					
Death	299 (15.1)	195 (12.1)	91 (28.0)	13 (29.5)	<0.001

Follow-up of study population.

^a^ Some patients received both coronary artery bypass graft and percutaneous coronary intervention.

^b^ One patient with mild and 14 patients with moderate valvular heart disease received valve repair or replacement during follow-up due to multivalvular disease, increase in severity, or combined with coronary artery bypass grafting.

CABG, coronary artery bypass grafting; PCI, percutaneous coronary intervention; SAVR, surgical aortic valve replacement; SMVR, surgical mitral valve replacement; SPVR, surgical pulmonary valve replacement; STVR, surgical tricuspid valve replacement; TAVR, transcatheter aortic valve replacement; TEER, transcatheter edge-to-edge repair; VHD, valvular heart disease.

**Figure 1:**
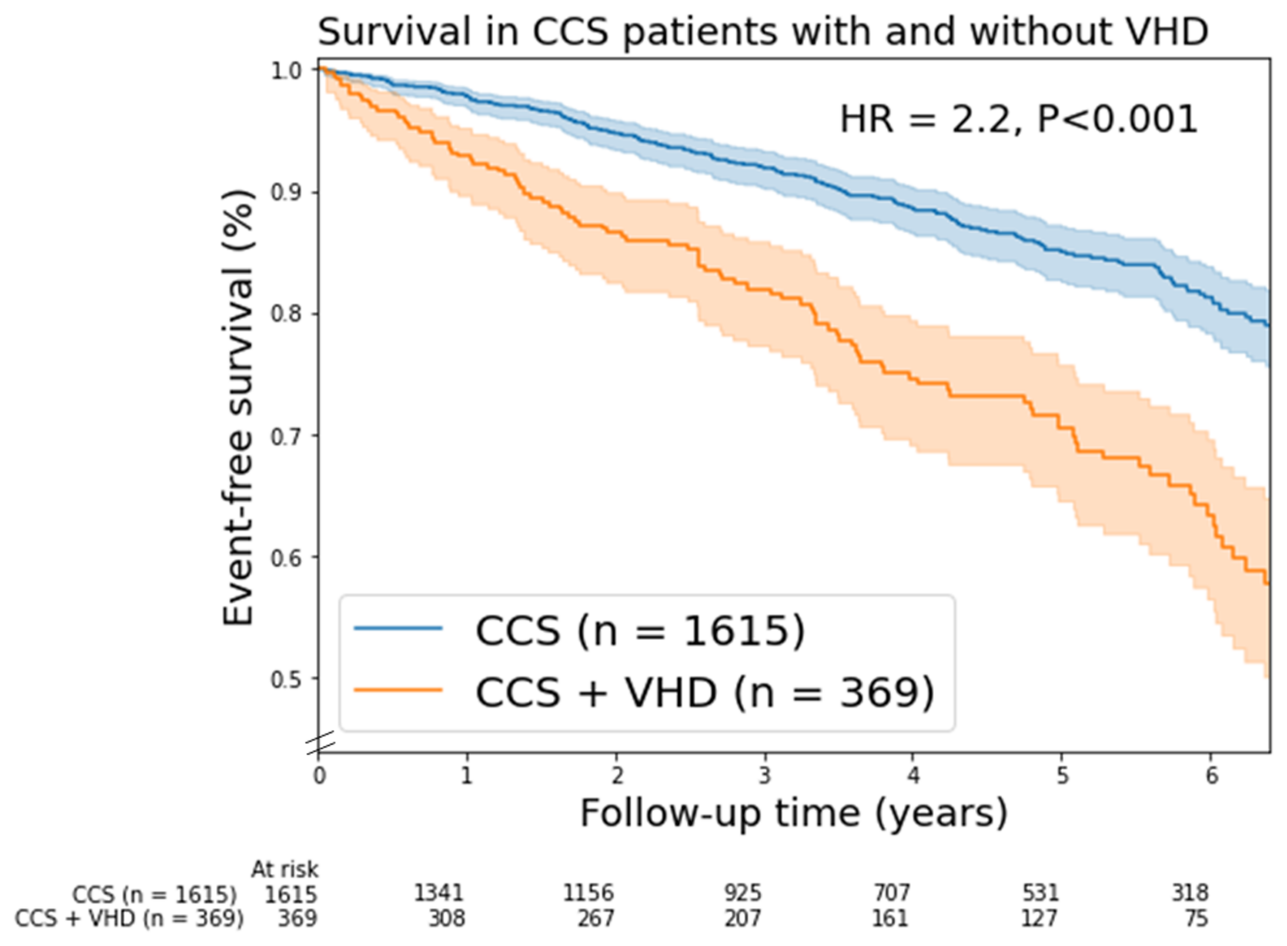
Kaplan-Meier mortality curves for chronic coronary syndrome (CCS) patients with/without valvular heart disease (VHD). In this figure, VHD is moderate and severe combined VHD. Shaded areas represent the 95% confidence intervals. HR, unadjusted hazard ratio.

A total of 299 patients (15%) died, of which 91 patients had moderate VHD and 13 patients had severe VHD. The mortality curves of patients with moderate and severe VHD showed a significant overlap and had both higher mortality rates compared to those with no/mild VHD (Supplementary Figure S2). Based on the overlap in the mortality curves and low number of patients with severe VHD (*n* = 44), it was decided to combine moderate and severe in further analysis (Figure 1).

### 3.4. Determinants of mortality

In univariable analysis, a number of variables were associated with mortality, as shown in Table 2. In multivariable analysis, moderate and severe combined VHD [hazard ratio (HR) = 1.33; 95% CI 1.02–1.72] remained associated with mortality independent of age (HR = 1.04; 95% CI 1.02–1.05), diabetes (HR = 1.58; 95% CI 1.24–2.00), current or former smoking (HR = 1.48; 95% CI 1.16–1.88), valve repair or replacement (HR = 1.50; 95% CI 1.02–2.20), COPD (HR = 1.96; 95% CI 1.42–2.70), chest pain (HR = 0.68; 95% CI 0.54–0.85), impaired renal function (HR = 1.61; 95% CI 1.27–2.04) and LV dysfunction (HR = 1.96; 95% CI 1.55–2.48). Details are shown in Table 2 and Supplementary Figure S3.

**Table 2:** Univariable and multivariable Cox regression analysis of variables associated with mortality: moderate and severe combined.

Variable	Univariable analysis		Multivariable analysis	
	HR (95% CI)	*P*-value	HR (95% CI)	*P*-value
Age per year	1.1 (1–1.1)	<0.001	1.04 (1.02–1.05)	<0.001
Male	1.3 (1–1.7)	0.025		
Hypertension	1.4 (1.1–1.8)	0.0041		
Diabetes	1.5 (1.2–2)	<0.001	1.58 (1.24–2.00)	<0.001
Dyslipidaemia	0.98 (0.77–1.2)	0.85		
Current or former smoker	1.4 (1.1–1.7)	0.0083	1.48 (1.16–1.88)	0.001
Family history of CAD	0.63 (0.48–0.83)	0.0011		
Myocardial infarction	1.5 (1.2–1.9)	<0.001		
PCI	1.2 (0.95–1.5)	0.13		
CABG	1.9 (1.4–2.5)	<0.001		
Valve repair or replacement	2.4 (1.7–3.5)	<0.001	1.50 (1.02–2.20)	0.04
Atrial fibrillation/-flutter	2 (1.5–2.6)	<0.001		
Stroke	1.6 (1–2.4)	0.032		
COPD	3 (2.2–4.1)	<0.001	1.96 (1.42–2.70)	<0.001
Chest pain	0.59 (0.47–0.75)	<0.001	0.68 (0.54–0.85)	0.001
Dyspnea	1.3 (1–1.7)	0.019		
Obesity	1.0 (0.8–1.3)	0.76		
Impaired renal function	2.5 (2.0–3.1)	<0.001	1.61 (1.27–2.04)	<0.001
Cholesterol per mmol/l	0.95 (0.85–1.1)	0.31		
LDL per mmol/l	0.86 (0.76–0.97)	0.018		
Triglyceride per mmol/l	1.1 (1–1.2)	0.031		
Left ventricular dysfunction	2.7 (2.1–3.4)	<0.001	1.96 (1.55–2.48)	<0.001
Moderate or severe VHD	2.2 (1.8–2.8)	<0.001	1.33 (1.02–1.72)	0.03

Univariable and multivariable Cox analysis. Variables with a *p*-value of *p* ≤ 0.05 on univariable mortality analysis were entered into the multivariable cox analysis with backward selection procedure. Moderate or severe VHD was a significant determinant of mortality independent of left ventricular dysfunction and other covariables.

CABG, coronary artery bypass grafting; CI, confidence interval; COPD, chronic obstructive pulmonary disease; HR, hazard ratio; LDL, low-density lipoprotein cholesterol; PCI, percutaneous coronary intervention; VHD, valvular heart disease.

Moderate VHD was associated with increased mortality (HR = 1.4; 95% CI 1.05–1.78), while severe VHD did not show a significant association with mortality (HR = 0.99; 95% CI 0.52–1.87, Supplementary Table S4).

### 3.5. Mortality and multivalvular disease

Patients with multivalvular disease (unadjusted HR = 3.2; 95% CI 2.4–4.9), had a higher mortality rate than patients with single valve heart disease (unadjusted HR = 1.9; 95% CI 1.5–2.5) and the group with no/mild VHD (Log-Rank *p* < 0.005, Supplementary Figure S4).

### 3.6. Mortality by VHD subtype

**Figure 2:**
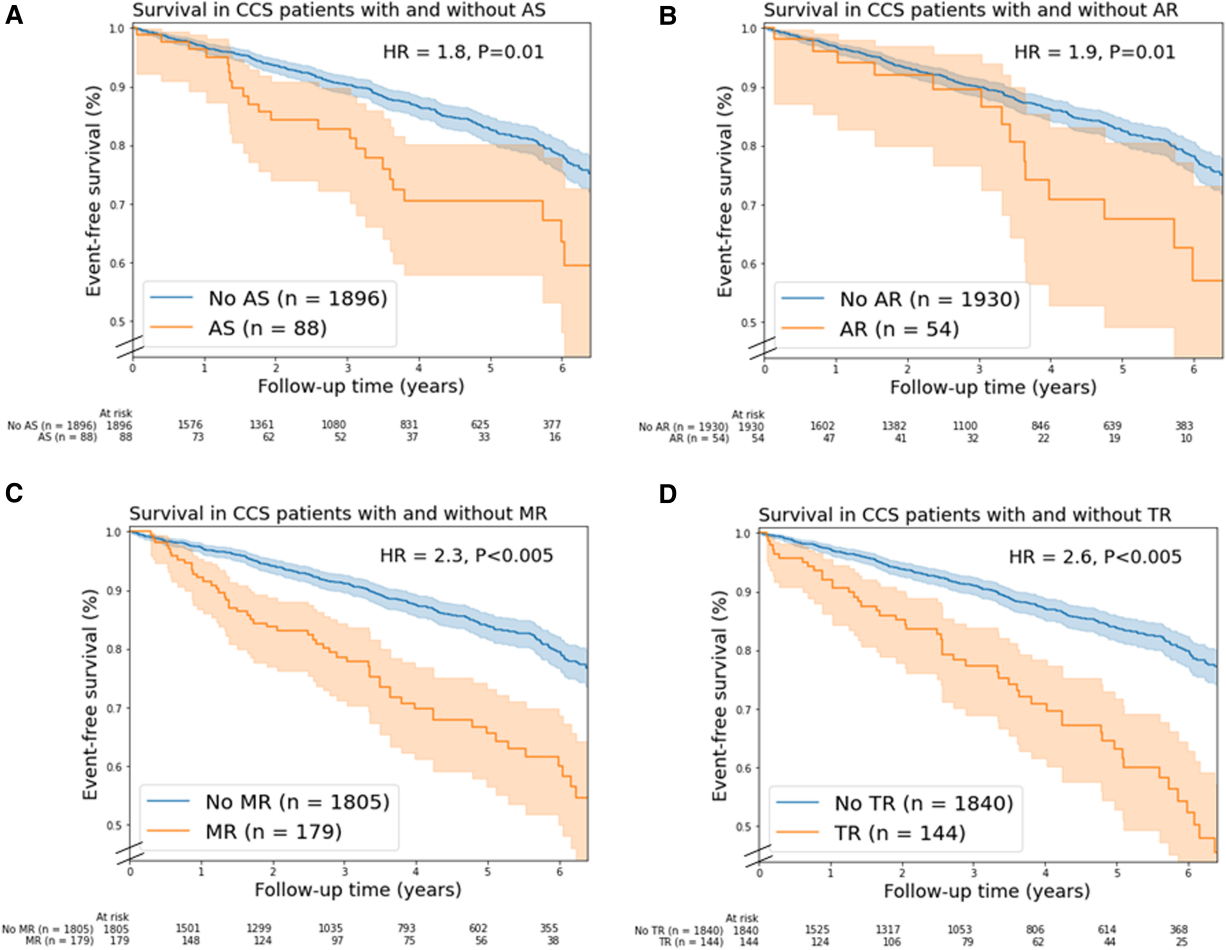
Kaplan-Meier mortality curves for chronic coronary syndrome (CCS) patients with/without (**A**) aortic stenosis, (**B**) aortic regurgitation, (**C**) mitral regurgitation, and (**D**) tricuspid regurgitation. In this figure, valvular heart disease (VHD) is moderate and severe combined VHD. Shaded areas represent the 95% confidence intervals. AR, aortic regurgitation; AS, aortic stenosis; HR, unadjusted hazard ratio; MR, mitral regurgitation; TR, tricuspid regurgitation.

Moderate and severe combined AS (unadjusted HR = 1.8, 95% CI 1.2–2.7), AR (unadjusted HR = 1.9, 95% CI 1.1–3.1), MR (unadjusted HR = 2.3, 95% CI 1.7–3.1), TR (unadjusted HR = 2.6, 95% CI 1.9–3.5) were associated with mortality (Figure 2). In addition, moderate and severe combined MS (*n* = 6), PR (*n* = 7) and PS (*n* = 1) were associated with mortality. In multivariable analysis, only moderate TR remained a significant predictor of mortality (HR = 1.6; 95% CI 1.2–2.3, Supplementary Table S5).

## 4. Discussion

This study in patients with CCS showed that moderate or severe combined VHD was associated with mortality, independent of LV function and other covariables. In the 1,984 patients studied, patients with CCS and moderate or severe VHD had a higher risk of mortality compared to patients with CCS only, which increased with the number of valves affected. These findings demonstrate the importance of echocardiographic assessment of VHD, in addition to LV function, in CCS patients.

### 4.1. Valvular heart disease in patients with chronic coronary syndrome

To our knowledge, this is the first study that investigates the prognostic value of VHD in patients with CCS. Severe VHD is an established determinant of mortality for which valve repair or replacement is recommended by current guidelines (18, 19). We observed that severe VHD was not an independent determinant of mortality in patients with CCS. This finding may be explained by the number of patients with severe VHD (*n* = 44) that may have been too small to detect a significant effect. Moreover, a substantial proportion of CCS patients with severe VHD received a valvular intervention during follow-up (55%) which likely reduced their mortality risk.

We found that moderate VHD was independently associated with mortality. Potential benefit of intervention on mortality for patients with moderate VHD is currently subject of debate (20). Ongoing studies are investigating the impact of both moderate AS and LV dysfunction (21). The hypothesis that TAVR improves outcomes in these patients is currently being prospectively investigated in the TAVR UNLOAD trial (22). Further prospective investigations are warranted to confirm the prognostic value of VHD in patients with moderate CCS and evaluate the impact of early intervention of VHD on mortality (23). Our findings support the need for improvement of care in VHD patients, which might be achieved by early valve repair or replacement. However, the complexity of the interplay between CCS and VHD on symptoms (24), cardiac damage (25, 26), and clinical course (27) make appropriate timing of intervention difficult (28).

Previous studies have investigated the association between moderate to severe TR and mortality. In 85%–90% of the patients, TR is caused secondary by left-sided heart failure (18). We found that moderate TR was independently associated with mortality in patients with CCS. Several studies have confirmed that TR is a predictor of mortality independent of LV dysfunction, pulmonary pressures, and right ventricle dilatation and dysfunction (29–31). Our findings suggest that TR is a marker of advanced disease in patients with CCS, which has more value than merely reflecting the severity of right/left ventricular dysfunction and pulmonary hypertension (29).

In this study in two tertiary centers, a significantly higher number of CCS patients had moderate or severe AS (*n* = 88), TR (*n* = 144), and MR (*n* = 179) compared to the general population with the same or older age (32–34). These relatively high VHD rates were anticipated in these specialized care centers, in which more complex medical conditions are seen. Lower rates of VHD in CCS patients may be expected in non-tertiary centers.

### 4.2. Pathophysiology

Our study shows that VHD has an incremental prognostic role in patients with CCS, which may have several explanations. Firstly, our findings demonstrate that patients with both CCS and moderate or severe VHD have more often risk factors for mortality, including older age, COPD, atrial fibrillation/-flutter, and a lower eGFR. Secondly, patients with both CCS and VHD may have more advanced cardiovascular calcifications, which are observed in atherosclerotic plaque formation, mitral annular calcification, and aortic artery calcification (4). These calcifications are strong determinants of cardiovascular events (35). Thirdly, both CCS and VHD can cause LV dysfunction through ischemia and LV remodeling, which may accelerate detoriation of the LV function leading to end-stage heart failure (7). Further longitudinal research is necessary to investigate the pathophysiological mechanisms of VHD in patients with CCS.

### 4.3. Clinical implications

The observed independent prognostic value of VHD suggests that it could have a crucial role in the non-invasive risk stratification of patients with CCS. However, despite the advantages of TTE, such as low-costs, portability and absence of radiation (36), TTE may not be performed in all patients with CCS as recommended in current guidelines. A recent study by Neglia et al. (37) showed that the diagnostic process was not according to the ESC guideline in 44% of the patients with CCS. This finding may have detrimental implications for patients with (suspected) CCS since undiagnosed and untreated VHD is associated with heart failure and mortality (34). Therefore, echocardiography should be performed in all patients with (suspected) CCS to rule out VHD and other cardiac diseases (4, 38).

The LV function is currently the only recommended echocardiographic assessed feature for risk stratification in patients with CCS. The results of this study indicate that comprehensive echocardiographic assessment of VHD should be included in the standard clinical workup of patients with CCS, at least in those with a normal LV function.

### 4.4. Study limitations

Several remarks can be made about this study. Firstly, the study had a retrospective cohort study design that has inherent limitations. Secondly, to minimize the amount of missing data, information was extracted from textual notes in electronic medical records. The cause of mortality was not available for all patients and was therefore not further differentiated. Thirdly, valve calcifications and quantitative parameters of echocardiography were not evaluated in this study. Fourthly, there may have been a selection bias in this study as the patients who underwent TTE may have had a higher a-priori risk of VHD, which could have influenced the results. Nevertheless, the included patients reflect a real-life population that were seen at a tertiary center.

## 5. Conclusions

VHD was an independent determinant of mortality in patients with CCS. This finding demonstrates the need for a comprehensive echocardiographic assessment of VHD, in addition to LV function, in CCS patients. Moreover, it indicates that complete assessment of VHD should be included in the standard clinical workup of patients with CCS to improve risk stratification.

## Data Availability

The original contributions presented in the study are included in the article/**Supplementary Material**, further inquiries can be directed to the corresponding author.
