# Supplementary material for: The impact of valvular heart disease in patients with chronic coronary syndrome
Source: Front Cardiovasc Med. 2023 Jul 21;10:1211322. doi: 10.3389/fcvm.2023.1211322 (PMC10401435; doi:10.3389/fcvm.2023.1211322)
Supplement: Supplementary file 1 [file Table1.docx]

**Supplementary Tables**

| **Supplementary Table S1. Data availability** | |
| --- | --- |
| **Characteristics** | **All patients (n=1984)** |
| Follow-up (years), n (%) | 1984 (100%) |
| Age (years), n (%) | 1984 (100%) |
| Male, n (%) | 1984 (100%) |
| Hypertension, n (%) | 1984 (100%) |
| Diabetes, n (%) | 1984 (100%) |
| Dyslipidaemia, n (%) | 1984 (100%) |
| Current or former smoker, n (%) | 1984 (100%) |
| Family history of CAD, n (%) | 1984 (100%) |
| Myocardial infarction, n (%) | 1984 (100%) |
| PCI, n (%) | 1984 (100%) |
| CABG, n (%) | 1984 (100%) |
| SAVR , n (%) | 1984 (100%) |
| TAVR, n (%) | 1984 (100%) |
| SMVR, n (%) | 1984 (100%) |
| TMVR, n (%) | 1984 (100%) |
| STVR, n (%) | 1984 (100%) |
| SPVR, n (%) | 1984 (100%) |
| Atrial fibrillation/-flutter, n (%) | 1984 (100%) |
| Stroke, n (%) | 1984 (100%) |
| COPD, n (%) | 1984 (100%) |
| Chest pain, n (%) | 1984 (100%) |
| Dyspnea, n (%) | 1984 (100%) |
| Other cardiac symptoms, n (%) | 1984 (100%) |
| BMI (kg/m2), mean (SD) | 1634 (83%) |
| eGFR (ml/min/1,73 m2), mean (SD) | 1868 (94%) |
| Cholesterol (mmol/l), mean (SD) | 1497 (75%) |
| LDL (mmol/l), mean (SD) | 1231 (62%) |
| Triglyceride (mmol/l), mean (SD) | 1454 (73%) |
| Antiplatelet therapy, n (%) | 1984 (100%) |
| Anticoagulants, n (%) | 1984 (100%) |
| ACE-inhibitor/ARB, n (%) | 1984 (100%) |
| Beta-blockers, n (%) | 1984 (100%) |
| Nitrates or other antianginal drugs, n (%) | 1984 (100%) |
| Calcium antagonists, n (%) | 1984 (100%) |
| Diuretics, n (%) | 1984 (100%) |
| Statins, n (%) | 1984 (100%) |
| Insulin, n (%) | 1984 (100%) |
| Other oral diabetic drugs, n (%) | 1984 (100%) |
|  |  |

Availability of data. The total proportion of missing data was 3%. ACE = angiotensin-converting enzyme; ARB = angiotensin receptor blocker; CAD = coronary artery disease; eGFR = estimated glomerular filtration rate; LDL = low-density lipoprotein cholesterol; SAVR = surgical aortic valve replacement; SMVR = surgical mitral valve replacement; SPVR = surgical pulmonary valve replacement; STVR = surgical tricuspid valve replacement; TAVR = transcatheter aortic valve replacement; TEER = transcatheter edge-to-edge repair; VHD = valvular heart disease.

| **Supplementary Table S2. Baseline Characteristics of study population** | | | | |  |
| --- | --- | --- | --- | --- | --- |
| **Characteristics** | **All patients (n=1984)** | **No/mild VHD (n=1615)** | **Moderate VHD (n=325)** | **Severe VHD (n=44)** | **p-value** |
| Follow-up (years), median [Q1,Q3] | 3.49 [1.72, 5.62] | 3.49 [1.72, 5.64] | 3.46 [1.77, 5.60] | 2.87 [1.29, 5.25] | 0.501 |
| Age (years), median [Q1,Q3] | 65.00 [57.00, 73.00] | 64.00 [56.00, 71.00] | 72.00 [65.00, 80.00] | 76.50 [69.75, 82.50] | <0.001 |
| Male, n (%) | 1169 (58.9) | 959 (59.4) | 187 (57.5) | 23 (52.3) | 0.548 |
| **Risk factors** |  |  |  |  |  |
| Hypertension, n (%) | 1072 (54.0) | 845 (52.3) | 202 (62.2) | 25 (56.8) | 0.005 |
| Diabetes, n (%) | 516 (26.0) | 429 (26.6) | 77 (23.7) | 10 (22.7) | 0.494 |
| Dyslipidaemia, n (%) | 651 (32.8) | 526 (32.6) | 109 (33.5) | 16 (36.4) | 0.830 |
| Current or former smoker, n (%) | 695 (35.0) | 585 (36.2) | 105 (32.3) | 5 (11.4) | 0.002 |
| Family history of CAD, n (%) | 635 (32.0) | 559 (34.6) | 74 (22.8) | 2 (4.5) | <0.001 |
| **Medical History** |  |  |  |  |  |
| Myocardial infarction, n (%) | 510 (25.7) | 397 (24.6) | 104 (32.0) | 9 (20.5) | 0.015 |
| PCI, n (%) | 644 (32.5) | 516 (32.0) | 113 (34.8) | 15 (34.1) | 0.596 |
| CABG, n (%) | 207 (10.4) | 145 (9.0) | 60 (18.5) | 2 (4.5) | <0.001 |
| SAVR, n (%) | 55 (2.8) | 36 (2.2) | 16 (4.9) | 3 (6.8) | 0.007 |
| TAVR, n (%) | 9 (0.5) | 5 (0.3) | 3 (0.9) | 1 (2.3) | 0.062 |
| SMVR, n (%) | 17 (0.9) | 11 (0.7) | 5 (1.5) | 1 (2.3) | 0.182 |
| TEER, n (%) | 1 (0.1) | 0 (0.0) | 0 (0.0) | 1 (2.3) | <0.001 |
| STVR, n (%) | 6 (0.3) | 6 (0.4) | 0 (0.0) | 0 (0.0) | 0.503 |
| SPVR, n (%) | 1 (0.1) | 1 (0.1) | 0 (0.0) | 0 (0.0) | 0.892 |
| Atrial fibrillation/-flutter, n (%) | 246 (12.4) | 131 (8.1) | 93 (28.6) | 22 (50.0) | <0.001 |
| Stroke, n (%) | 111 (5.6) | 82 (5.1) | 27 (8.3) | 2 (4.5) | 0.066 |
| COPD, n (%) | 128 (6.5) | 90 (5.6) | 31 (9.5) | 7 (15.9) | 0.001 |
| **Clinical examination** |  |  |  |  |  |
| Chest pain, n (%) | 1168 (58.9) | 986 (61.1) | 164 (50.5) | 18 (40.9) | <0.001 |
| Dyspnea, n (%) | 606 (30.5) | 471 (29.2) | 116 (35.7) | 19 (43.2) | 0.012 |
| Other cardiac symptoms, n (%) | 418 (21.1) | 340 (21.1) | 72 (22.2) | 6 (13.6) | 0.429 |
| BMI (kg/m2), mean (SD) | 27.45 (5.39) | 27.66 (5.40) | 26.64 (5.28) | 26.26 (5.19) | 0.007 |
| **Laboratory parameters** |  |  |  |  |  |
| eGFR (ml/min/1,73 m2)^a^, mean (SD) | 70.05 (20.92) | 72.02 (20.53) | 61.71 (20.22) | 62.07 (23.31) | <0.001 |
| Cholesterol (mmol/l), mean (SD) | 4.56 (1.27) | 4.58 (1.26) | 4.52 (1.36) | 4.30 (1.27) | 0.495 |
| LDL (mmol/l), mean (SD) | 2.56 (1.13) | 2.58 (1.12) | 2.48 (1.15) | 2.45 (0.97) | 0.450 |
| Triglyceride (mmol/l), mean (SD) | 1.62 (1.09) | 1.64 (1.09) | 1.51 (1.10) | 1.37 (0.91) | 0.113 |
| **Baseline medication** |  |  |  |  |  |
| Antiplatelet therapy, n (%) | 1212 (61.1) | 998 (61.8) | 191 (58.8) | 23 (52.3) | 0.285 |
| Anticoagulants, n (%) | 294 (14.8) | 176 (10.9) | 98 (30.2) | 20 (45.5) | <0.001 |
| ACE-inhibitor/ARB, n (%) | 901 (45.4) | 705 (43.7) | 172 (52.9) | 24 (54.5) | 0.004 |
| Beta-blockers, n (%) | 1035 (52.2) | 797 (49.3) | 214 (65.8) | 24 (54.5) | <0.001 |
| Nitrates or other antianginal drugs, n (%) | 555 (28.0) | 444 (27.5) | 102 (31.4) | 9 (20.5) | 0.192 |
| Calcium antagonists, n (%) | 584 (29.4) | 481 (29.8) | 97 (29.8) | 6 (13.6) | 0.067 |
| Diuretics, n (%) | 501 (25.3) | 364 (22.5) | 119 (36.6) | 18 (40.9) | <0.001 |
| Statins, n (%) | 1192 (60.1) | 946 (58.6) | 222 (68.3) | 24 (54.5) | 0.004 |
| Insulin, n (%) | 226 (11.4) | 177 (11.0) | 43 (13.2) | 6 (13.6) | 0.448 |
| Other oral diabetic drugs, n (%) | 365 (18.4) | 308 (19.1) | 50 (15.4) | 7 (15.9) | 0.268 |

Values are mean (standard deviation), n (%), or median [interquartile range]. ^a^ Calculated with the Chronic Kidney Disease Epidemiology Collaboration (CKD-EPI) creatinine equation (13). ACE = angiotensin-converting enzyme; ARB = angiotensin receptor blocker; CAD = coronary artery disease; eGFR = estimated glomerular filtration rate; LDL = low-density lipoprotein cholesterol; SAVR = surgical aortic valve replacement; SMVR = surgical mitral valve replacement; SPVR = surgical pulmonary valve replacement; STVR = surgical tricuspid valve replacement; TAVR = transcatheter aortic valve replacement; TEER = transcatheter edge-to-edge repair; VHD = valvular heart disease.

| **Supplementary Table S3. Echocardiography characteristics of study population** | | | | |
| --- | --- | --- | --- | --- |
|  | **All patients (n=1984)** | **No/mild VHD (n=1615)** | **Moderate VHD (n=325)** | **Severe VHD (n=44)** |
| **Left ventricular function, n (%)** |  |  |  |  |
| Normal | 1479 (74.5) | 1258 (77.9) | 197 (60.6) | 24 (54.5) |
| Mildly impaired | 310 (15.6) | 230 (14.2) | 69 (21.2) | 11 (25.0) |
| Moderately impaired | 156 (7.9) | 105 (6.5) | 44 (13.5) | 7 (15.9) |
| Severely impaired | 39 (2.0) | 22 (1.4) | 15 (4.6) | 2 (4.5) |
| **Aortic stenosis grade, n (%)** |  |  |  |  |
| Normal/mild | 1896 (95.6) | 1615 (100.0) | 262 (80.6) | 19 (43.2) |
| Moderate | 64 (3.2) | 0 (0.0) | 63 (19.4) | 1 (2.3) |
| Severe | 24 (1.2) | 0 (0.0) | 0 (0.0) | 24 (54.5) |
| **Aortic regurgitation grade, n (%)** |  |  |  |  |
| Normal/mild | 1930 (97.3) | 1615 (100.0) | 279 (85.8) | 36 (81.8) |
| Moderate | 50 (2.5) | 0 (0.0) | 46 (14.2) | 4 (9.1) |
| Severe | 4 (0.2) | 0 (0.0) | 0 (0.0) | 4 (9.1) |
| **Mitral stenosis grade, n (%)** |  |  |  |  |
| Normal/mild | 1978 (99.7) | 1615 (100.0) | 320 (98.5) | 43 (97.7) |
| Moderate | 6 (0.3) | 0 (0.0) | 5 (1.5) | 1 (2.3) |
| Severe | 0 (0.0) | 0 (0.0) | 0 (0.0) | 0 (0.0) |
| **Mitral regurgitation grade, n (%)** |  |  |  |  |
| Normal/mild | 1805 (91.0) | 1615 (100.0) | 164 (50.5) | 26 (59.1) |
| Moderate | 176 (8.9) | 0 (0.0) | 161 (49.5) | 15 (34.1) |
| Severe | 3 (0.2) | 0 (0.0) | 0 (0.0) | 3 (6.8) |
| **Tricuspid regurgitation grade, n (%)** |  |  |  |  |
| Normal/mild | 1840 (92.7) | 1615 (100.0) | 204 (62.8) | 21 (47.7) |
| Moderate | 128 (6.5) | 0 (0.0) | 121 (37.2) | 7 (15.9) |
| Severe | 16 (0.8) | 0 (0.0) | 0 (0.0) | 16 (36.4) |
| **Pulmonary stenosis grade, n (%)** |  |  |  |  |
| Normal/mild | 1983 (99.9) | 1615 (100.0) | 325 (100.0) | 43 (97.7) |
| Moderate | 0 (0.0) | 0 (0.0) | 0 (0.0) | 0 (0.0) |
| Severe | 1 (0.1) | 0 (0.0) | 0 (0.0) | 1 (2.3) |
| **Pulmonary regurgitation grade, n (%)** |  |  |  |  |
| Normal/mild | 1977 (96,6) | 1615 (100.0) | 320 (98.5)) | 42 (95.5) |
| Moderate | 7 (0.4) | 0 (0.0) | 5 (1.5) | 2 (4.5) |
| Severe | 0 (0.0) | 0 (0.0) | 0 (0.0) | 0 (0.0) |

VHD = valvular heart disease.

| **Supplementary Table S4. Univariable and multivariable Cox regression analysis of variables associated with mortality: moderate and severe analysis** | | | | |
| --- | --- | --- | --- | --- |
| **Variable** | **Univariable analysis** | | **Multivariable analysis** | |
|  | **HR (95% CI)** | **P-value** | **HR (95% CI)** | **P-value** |
| No/mild VHD | 1 [Reference] | NA | 1 [Reference] | NA |
| Moderate VHD | 2.2 (1.7-2.8) | <0.001 | 1.4 (1.05-1.78) | 0.02 |
| Severe VHD | 2.8 (1.6-4.8) | <0.001 | 0.99 (0.52-1.87) | 0.96 |
|  |  |  |  |  |

*Model was adjusted for age, diabetes, current or former smoking, valve repair or replacement, chronic obstructive pulmonary disease, chest pain, impaired renal function, and LV dysfunction. CI = confidence interval; HR = hazard ratio; VHD = valvular heart disease.

| **Supplementary Table S5. Univariable and multivariable Cox regression analysis of variables associated with mortality: valvular heart disease subtype analysis** | | | | |
| --- | --- | --- | --- | --- |
| **Variable** | **Univariable analysis** | | **Multivariable analysis** | |
|  | **HR (95% CI)** | **P-value** | **HR (95% CI)** | **P-value** |
| No/mild AS | 1 [Reference] | NA | 1 [Reference] | NA |
| Moderate AS | 1.5 (0.9-2.6) | 0.09 |  |  |
| Severe AS | 2.5 (1.3-5.2) | 0.009 |  |  |
| AR |  |  |  |  |
| No/mild AR | 1 [Reference] | NA | 1 [Reference] | NA |
| Moderate AR | 1.8 (1.04-3.02) | 0.04 |  |  |
| Severe AR | 3.4 (0.9-13.8) | 0.08 |  |  |
| MR |  |  |  |  |
| No/mild MR | 1 [Reference] | NA | 1 [Reference] | NA |
| Moderate MR | 2.3 (1.7-3.0) | <0.001 |  |  |
| Severe MR | 4.2 (0.6-30.3) | 0.15 |  |  |
| TR |  |  |  |  |
| No/mild TR | 1 [Reference] | NA | 1 [Reference] | NA |
| Moderate TR | 2.6 (1.9-3.6) | <0.001 | 1.6 (1.2-2.3) | 0.005 |
| Severe TR | 2.5 (0.9-6.7) | 0.07 | 1.2 (0.3-2.4) | 0.8 |
|  |  |  |  |  |

*Model was adjusted for age, diabetes, current or former smoking, valve repair or replacement, chronic obstructive pulmonary disease, chest pain, impaired renal function, and LV dysfunction. AR = aortic regurgitation; AS = aortic stenosis; CI = confidence interval; MR = mitral regurgitation; NA = not applicable; TR = tricuspid regurgitation; VHD = valvular heart disease.
