# Supplementary material for: The impact of valvular heart disease in patients with chronic coronary syndrome
Source: Front Cardiovasc Med. 2023 Jul 21;10:1211322. doi: 10.3389/fcvm.2023.1211322 (PMC10401435; doi:10.3389/fcvm.2023.1211322)

**Supplementary Figures**

**Supplementary Figure S1. Inclusion flowchart*.* CCS = chronic coronary syndrome; EHR = electronic health records; TTE = transthoracic echocardiography.**


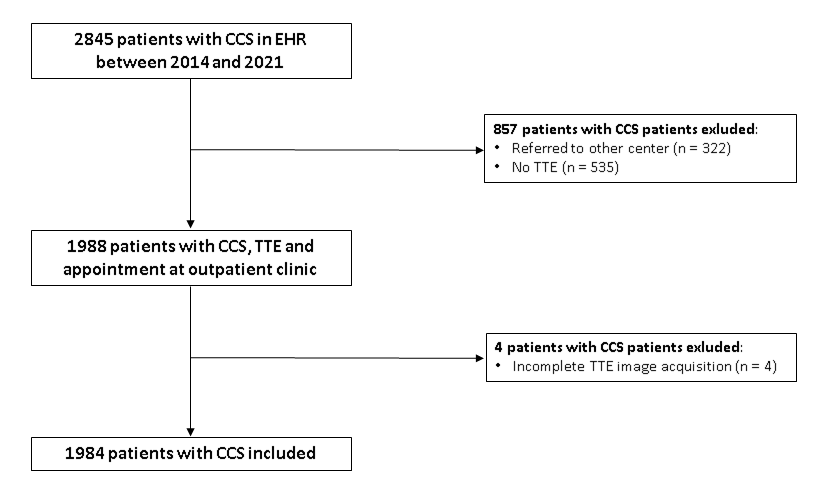


**Supplementary Figure S2. Kaplan-Meier mortality curves for patients with chronic coronary syndrome stratified by severity of valvular heart disease (VHD). There is a considerable overlap between the mortality curves of patients with moderate and severe valvular heart disease. Shaded areas represent the 95% confidence intervals.**


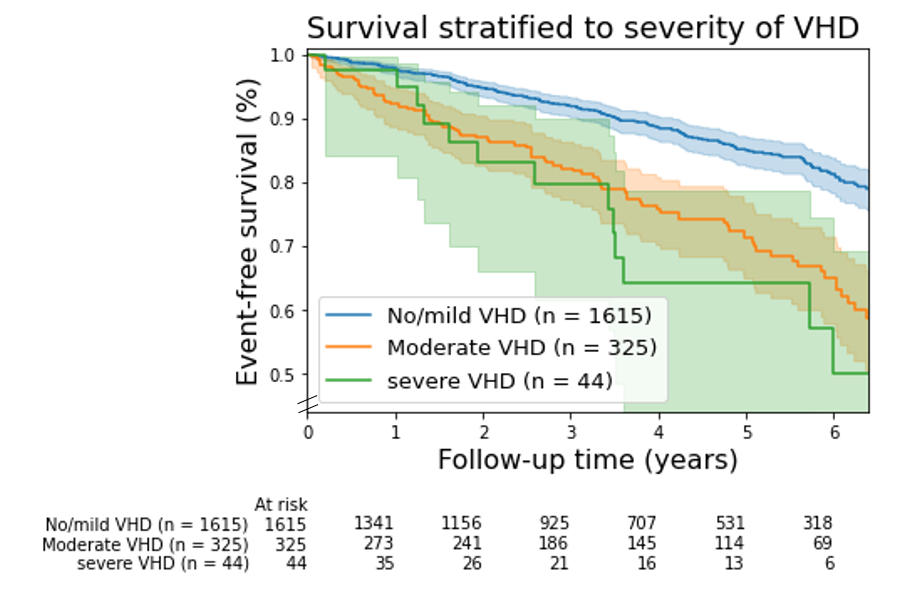


**Supplementary Figure S3. Kaplan-Meier mortality curves for patients with chronic coronary syndrome stratified by left ventricular dysfunction (LVD) and valvular heart disease (VHD). In this figure, VHD is moderate and severe combined VHD. Patients with VHD and LVD have higher mortality rates compared to patients with LVD only (Log-Rank p=0.01). Shaded areas represent the 95% confidence intervals.**


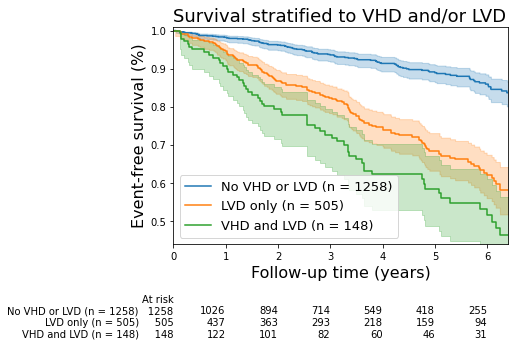


**Supplementary Figure S4. Kaplan-Meier mortality curves for patients with chronic coronary syndrome stratified by the number of valves affected. In this figure, valvular heart disease (VHD) is moderate and severe combined VHD. Shaded areas represent the 95% confidence intervals.**


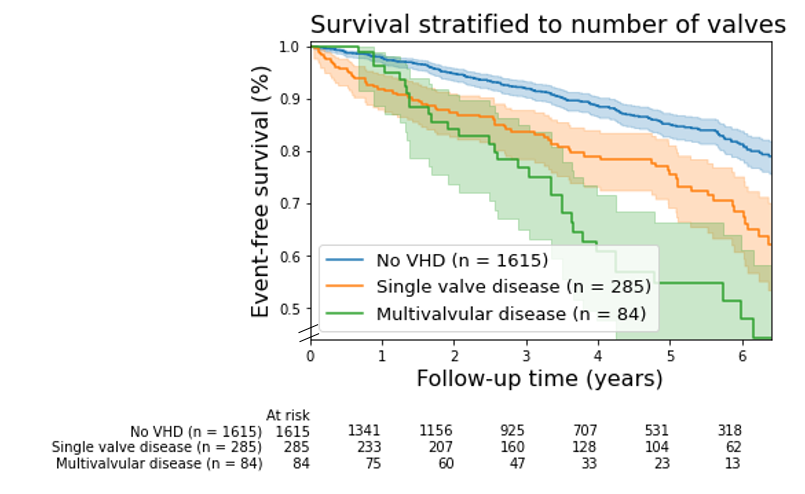

Supplement: Supplementary file 2 [file Datasheet1.docx]
